# Geometrical Microfeature Cues for Directing Tubulogenesis of Endothelial Cells

**DOI:** 10.1371/journal.pone.0041163

**Published:** 2012-07-19

**Authors:** Yifeng Lei, Omar F. Zouani, Murielle Rémy, Cédric Ayela, Marie-Christine Durrieu

**Affiliations:** 1 INSERM U1026, Université Victor Segalen Bordeaux 2, Bordeaux, France; 2 CBMN, UMR CNRS 5248, Université Bordeaux 1, Pessac, France; 3 IMS, UMR CNRS 5218, Université de Bordeaux, Talence, France; Johns Hopkins University, United States of America

## Abstract

Angiogenesis, the formation of new blood vessels by sprouting from pre-existing ones, is critical for the establishment and maintenance of complex tissues. Angiogenesis is usually triggered by soluble growth factors such as VEGF. However, geometrical cues also play an important role in this process. Here we report the induction of angiogenesis solely by SVVYGLR peptide micropatterning on polymer surfaces. SVVYGLR peptide stripes were micropatterned onto polymer surfaces by photolithography to study their effects on endothelial cell (EC) behavior. Our results showed that the EC behaviors (cell spreading, orientation and migration) were significantly more guided and regulated on narrower SVVYGLR micropatterns (10 and 50 µm) than on larger stripes (100 µm). Also, EC morphogenesis into tube formation was switched on onto the smaller patterns. We illustrated that the central lumen of tubular structures can be formed by only one-to-four cells due to geometrical constraints on the micropatterns which mediated cell-substrate adhesion and generated a correct maturation of adherens junctions. In addition, sprouting of ECs and vascular networks were also induced by geometrical cues on surfaces micropatterned with SVVYGLR peptides. These micropatterned surfaces provide opportunities for mimicking angiogenesis by peptide modification instead of exogenous growth factors. The organization of ECs into tubular structures and the induction of sprouting angiogenesis are important towards the fabrication of vascularized tissues, and this work has great potential applications in tissue engineering and tissue regeneration.

## Introduction

Angiogenesis, the formation of new blood vessels by a process of sprouting from pre-existing ones [1], plays an important role in both normal developmental processes and numerous pathologies, ranging from tumor growth and metastasis to inflammation and ocular diseases [2]. It is also critical for the establishment and maintenance of large engineered tissues, and as known, vascularization is a critical challenge in tissue engineering [3]. Angiogenesis involves in multiple steps: degradation of the basement membrane, endothelial cell (EC) migration, proliferation, tube formation, and blood vessel maturation [4]. These steps are stimulated and controlled by a complex network of intracellular signaling mechanisms [5].

Ever since the introduction of the *in vitro* model of angiogenesis [1], many *in vitro* and *in vivo* assays have been developed to study and follow the sophisticated process of angiogenesis [6], [7]. *In vitro*, angiogenesis is often studied by stimulating a monolayer of endothelial cells to assemble into tubes and sprouting [8]. Specific angiogenic molecules can initiate the process of angiogenesis and specific inhibitory molecules can stop it [5]. To promote local angiogenesis, one major theme is the delivery of angiogenic molecules. Numerous inducers of angiogenesis have been identified, such as extracellular matrix (ECM) proteins (laminin, collagen, etc) [9] and growth factors (VEGF, bFGF, etc) [5], [10]. Conventional tissue engineering strategies utilized some biological molecules mentioned above to promote angiogenesis [11].

Vascular endothelial growth factor (VEGF, or VEGF-A) is the most potent angiogenic protein described to date [5]. VEGF plays a key role in most morphogenetic events during angiogenesis [12]. Many studies have demonstrated that VEGF enhanced proliferation, migration, sprouting [13], [14] and tube formation of endothelial cells [12]. However, the design of VEGF therapy is costly, and one of the most critical problems associated with this therapy is the uncontrollable dose of VEGF delivered [15], which results in negative side effects in non-targeted tissues (hyperpermeable vessels, hypotension, stimulation of tumor growth, and uncontrolled neovascularization) [16].

Otherwise, sequence of angiogenic factors in ECM proteins can be mimicked closely in the process of angiogenesis. The main motivation for developing new synthetic mimicking culture systems is to minimize utilization of the above-mentioned natural ECM proteins and growth factors with the aim of reducing cost and avoiding biological challenges in purification and validation [17]. Numerous reports have described biological activities of ECM-derived peptides corresponding to active sites in proteins, thus using them for triggering angiogenesis [18], [19]. Peptides offer advantages over the use of their parent ECM proteins: their chemical definition, accessibility, stability, practicality and simplicity to be conjugated with materials in order to mimic *in vivo* microenvironment [17], [20].

Among peptides investigated, a powerful candidate that induces angiogenesis is SVVYGLR peptide sequence. This peptide is a novel binding motif that was found adjacent to the RGD sequence in the osteopontin molecule following thrombin cleavage [21]. Previous studies reported that soluble SVVYGLR peptides activated adhesion, migration of endothelial cells *in vitro* [18], and induced angiogenesis *in vivo* in its soluble form [22]. SVVYGLR peptides are also shown to promote neovascularization in artificial bone marrow scaffold biomaterials [23]. It was reported to have much stronger angiogenic activity as compared with VEGF [18], [23]. SVVYGLR presented in previous works were either coated on the surfaces or dissolved in solution for induction of local angiogenesis. However, the study of angiogenesis process and characteristics in these systems were still difficult because of the inaccessibility to this local microenvironment. To simply the complexity of numerous variables typical for ECs’ native microenvironment, advanced synthetic systems could greatly facilitate the study of angiogenesis process.

Herein, our strategy consists in the use of microengineering tools to generate materials micropatterned with angiogenic biomolecules on their surfaces to control the cell behaviors. Microengineering technologies provide powerful tools to study *in vitro* cell-microenvironment interactions [24]. They allow the control of the presentation of angiogenic biomolecules on surfaces in pre-decided sizes and shapes, thus influencing cell placement, orientation, morphology, and cell functions on the surfaces [24]. Microengineered surfaces for cell-based assay were developed to control cell shape and behaviors as previously reported [19], [25]–[30].

In this study, we focus on the covalent grafting of SVVYGLR peptides onto polymer surfaces with controlling geometries, and we aim to study their effect on EC behaviors as well as angiogenesis. Different micropatterns of SVVYGLR peptides on polymer surfaces were prepared by photolithographic technique. The EC behaviors, the induction of EC tube formation, and the vascular network formation on the micropatterned surfaces were addressed. We observe that the EC behaviors were significantly more guided and tube formation was switched on onto narrower micropatterns (10 and 50 µm) as compared with larger stripes (100 µm). We illustrate that the central lumen of tubular structure can be formed solely by geometrical cues. Then, we report that only single-to-four cells can form central lumen due to geometrical constraints on the micropatterns which mediated cell-substrate adhesion and generated a correct maturation of adherens junctions (AJs) [31]. In addition, sprouting angiogenesis of ECs and vascular networks were also induced by geometrical cues on surfaces micropatterned with SVVYGLR peptides. These findings serve to identify mechanism characteristics that alter EC lumen formation and sprouting in angiogenesis process, which may be utilized for innovating biomaterials and for application in tissue engineering.

## Materials and Methods

### Materials

Polyethylene terephthalate (PET) film is a commercial film obtained from Goodfellow, France. Inorganic reagents (NaOH, KMnO_4_, H_2_SO_4_, and HCl), acetone, acetonitrile, dimethylaminopropyl-3-ethylcarbodiimide hydrochloride (EDC), N-hydroxy Succinimide (NHS) and 2-(N-morpholino)-ethanesulfonic acid (MES) were purchased from Sigma-Aldrich, France. GDSVVYGLR peptides and GDSVVYGLRK-FITC fluorescent peptides were synthesized by Genecust, France.

### Covalent Grafting of Peptides onto PET Surface

PET surfaces were modified according to Chollet et al. [32] with some modifications. Briefly, PET was hydrolyzed and oxidized in order to create COOH groups on the surface (labelled as “PET-COOH”). Then, the surfaces were immersed in a solution of EDC (0.2 M) + NHS (0.1 M) + MES (0.1 M) in MilliQ water for activation. Subsequently, the surfaces were immersed in peptide solution (GDSVVYGLR peptides dissolved in PBS with a concentration of 10^−3 ^M) for 16 h at room temperature for peptide immobilization onto PET surfaces (named as “SVVYGLR”). This peptide concentration was chosen after preliminary cell alignment tests on micropatterned surfaces with 50 µm peptide stripes (Fig. S1). After covalent immobilization, the surfaces were rinsed with MilliQ water for 1 week in order to remove the physically adsorbed peptides.

### Preparation of Micropatterned Surfaces

Micropatterns on polymer surfaces were fabricated by photolithographic technique as previously developed [33]. Briefly, the surfaces of materials were coated with S1818 photoresist (Rohm and Haas, USA) and spun at 3000 rpm for 30 s to obtain a uniform photoresist layer with a thickness of approximately 2 µm. The surfaces were baked at 115°C for 1 min for drying. The surfaces were then exposed to UV light (60 W) through a high-resolution Cr mask with predesigned pattern dimensions (Femto-St Sciences & Technologies, France) for 18 s. Subsequently the surfaces were developed in Microposit Developer solution (Rohm and Haas, USA) for 40 s to dissolve the exposed photoresist, resulting in the desired pattern on material surfaces.

The micropatterns on surfaces were prepared after the “PET-COOH” step, subsequently the surface activation and peptide immobilization were realized as described in the above section. Finally, the photoresist surrounding the peptide micropatterns was removed by acetone, resulting in SVVYGLR peptide micropatterns on PET surfaces.

### Surface Characterization

X-ray photoelectron spectroscopy (XPS) was used to characterize the surface chemical composition during the process of peptide immobilization. XPS was characterized on a VG Scientific ESCALAB photoelectron spectrometer, with an MgK X-ray source (1253.6 eV photons, 100W). Spectra were referenced by setting carbon pollution at 284.8 eV.

Fluorescent peptides were employed to facilitate the visualization of peptide micropatterns according to Zouani et al. [34]. In this case, GDSVVYGLR peptides were covalently conjugated to FITC fluochromes via lysine (labeled as “GDSVVYGLRK-FITC”), and immobilized onto micropatterned PET surfaces as described in the above sections. Epifluorescence microscopy (Leica DM5500B, Germany) was employed for visualization of fluorescent peptide patterns on polymer surfaces.

### Cell Culture

Human umbilical vein endothelial cells (HUVECs) were obtained from the human umbilical cord vein according to the methods described previously [35], [36]. HUVECs were isolated and grown on gelatin coated culture flasks in HUVEC complete culture medium (IMDM (Invitrogen, France) supplemented with 20% (v/v) fetal bovine serum (FBS) (PAA, France) and 0.4% (v/v) EC growth supplement/heparin kit (Promocell, France)). Cells were subcultured using trypsin/EDTA (Invitrogen, France) and maintained in a humidified atmosphere containing 5% (v/v) CO_2_ at 37°C. Cells at passages 3 to 5 were used for experiments.

### Immobilization of SVVYGLR Peptides on EC Behaviors

To evaluate the effect of immobilization of SVVYGLR peptides on cell behaviors, cells were cultured on pristine PET surfaces (labeled as “PET”) and PET grafted with SVVYGLR peptides (labeled as “Grafted SVV”). HUVECs were seeded at a density of 50000 cells/cm^2^ in serum-free IMDM medium for 4 h on different surfaces (n = 6). Cells were allowed to adhere for 4 h, then IMDM medium was removed and ECs were cultured in IMDM medium containing 10% FBS for 1 and 3 days.

In competitive experiments, EC behaviors were also examined using soluble SVVYGLR peptides to ensure that improved EC adhesion and spreading were due to specific interaction with the SVVYGLR peptides that immobilized onto PET surfaces. ECs were seeded onto SVVYGLR peptides immobilized surfaces at 50000 cells/cm^2^ in serum-free IMDM medium containing soluble SVVYGLR peptides at 10, 100, 1000 and 10000 ng/mL (labeled as “+10 SVV”, “+100 SVV”, “+1000 SVV”, “+10000 SVV”, respectively). EC adhesion and spreading were evaluated after 4 h incubation.

### Immunofluorescent Staining

Immunofluorescent staining was performed to visualize the ECs on different surfaces. After cell culture, cells were fixed by 4% (w/v) paraformaldehyde (PFA), permeabilized with 0.5% Triton-X 100, blocked with 1% bovine serum albumin (BSA) in PBS solution, and stained with primary and secondary antibodies. The primary antibodies used were mouse anti-vinculin (Sigma, France), mouse anti-CD31 (PECAM-1) (Invitrogen, France). The secondary antibodies were anti-mouse antibodies conjugated with Alexa Fluor® 568 (Invitrogen, France). Cell actin cytoskeletons and nuclei were stained with Alexa Fluor® 488 phalloidin (Invitrogen, France) and DAPI (Sigma, France), respectively. The samples were mounted with ProLong® Gold antifade reagent (Invitrogen, France) and observed by fluorescence microscopy.

Cell adhesion and spreading were examined by ImageJ software (NIH, http://rsb.info.nih.gov/ij/). Cell nuclei were counted for evaluation of adherent cell number. The cell areas were determined by tracing the cell edges from actin cytoskeleton. At least 20 fields at low magnification (10 X) on each surface were analyzed for this study.

### Micropatterning of SVVYGLR Peptides on EC Behaviors

To evaluate the effects of micropatterning of peptides on EC functions, polymer surfaces micropatterned with 10, 50 and 100 µm SVVYGLR peptide stripes with same interspaces of 100 µm were prepared by photolithography (the surfaces were labeled as “10 µm”, “50 µm” and “100 µm”, respectively). Immobilization of SVVYGLR peptides onto polymer surfaces homogenously without patterning served as controls (labeled as “unpatterned”). ECs were seeded onto different surfaces at a density of 50000 cells/cm^2^ in EGM®-2 medium (Lonza, France), to study the effect of SVVYGLR peptide micropatterns on cellular functions.

### Cell Spreading, Orientation and Migration

ECs on patterned and unpatterned surfaces were fixed after 26 h of culture and immunofluorescently stained as described previously. Cell areas were evaluated by ImageJ software.

Cell orientation (alignment and elongation) were evaluated as previously demonstrated [37], [38]. The cell was fitted into an ellipse by ImageJ software. The cell body alignment angle, defined as the angle of the major axis of the cell body with respect to the direction of micropatterns [37], was measured using ImageJ. For cells on unpatterned surfaces, the angle of the cell body major axis with respect to an arbitrary axis (here fixed at 0°) was taken for cell alignment angle. A cell was aligned perfectly parallel to the direction of pattern when the alignment angle was 0° and perfectly perpendicular to the patterns when the alignment angle was 90°. Cell area (*s*) and perimeter (*l*) were measured by the “measure” tool in ImageJ, and cell morphology was characterized by cell shape index (*I*) which was calculated according to the following formula: *I = *4π*s*/*l* ^2^ [38]. Cells are round when *I* is equal to 1 and cells become infinitely elongated as *I* approaches 0. About eighty cells for each surface were analyzed in this study.

For monitoring cell migration, HUVECs were seeded onto patterned and unpatterned surfaces at a density of 50000 cells/cm^2^ and cultured in EGM®-2 medium at 37°C and 5% CO_2_. ECs were allowed to adhere and align onto SVVYGLR peptide micropatterns in incubator for 4 h. Then the samples were transferred to time-lapse microscopy (Leica DM5500B) in a humidified atmosphere containing 5% CO_2_ at 37°C, and cell migration on patterns was monitored by Leica MMAF software and automated stages. The images were photographed in intervals of every 6 min during 12 h. Then the videos were analyzed using the free software “Time Lapse Analyser” (TLA: http://www.informatik.uni-ulm.de/ni/staff/HKestler/tla/). For quantification of cell motility, cell trajectories, total distances and migration rate (µm/min) were calculated. A minimum of 30 cells per condition were analyzed. Experiments were done in duplicates for each surface.

### Quantification of Focal Adhesions and Adherens Junctions

To quantify the number and size of focal adhesions (FAs), the fluorescent images of vinculin staining were analyzed using ImageJ [32], [39]. The raw images were opened and converted into 8-bit file, smoothed by the unsharp mask feature (settings 1∶0.2) and background removed (rolling ball radius 10). The resulting images were then converted to binary images by setting a same threshold. The threshold values were determined empirically by selecting a setting which gave most accurate binary images. The cell area was determined by manual delineation on raw fluorescent images. The number of focal contacts per cell and mean contact area per cell were calculated by “analyse particles” in ImageJ, and contacts smaller than 3 pixels were not taken into account. A minimum of 30 cells per condition were analyzed.

For quantification of cell-cell adherens junctions, the cells on different substrates were stained with antibody against platelet endothelial cell adhesion molecule (PECAM-1). The fluorescence images of PECAM staining were processed in ImageJ software. AJs of ECs were obtained from PECAM staining and were binarized with same thresholds on different surfaces. The AJ size and density on each surface was quantified by measuring respective cell-cell junction width and junction density with the “plot profile” tool in ImageJ software [31].

### EC Morphogenesis into Tube-like Structure

HUVECs were seeded at a density of 50000 cells/cm^2^ in EGM®-2 medium, cell growth was maintained at 37°C and 5% CO_2_. The cells on surfaces were photographed by phase contrast microscopy (Zeiss Axiovert 25, Germany), and the cells were fixed when morphogenesis of ECs seemed to appear after about 26 h of culture.

ECs were labelled with Cell Tracker Green (CMFDA, 5 µM) (Invitrogen, France) for 30 minutes prior to fixation, according to the technical protocol of the product. The cells were then fixed by 4% PFA, counterstained with actin and DAPI and mounted for observation. Confocal microscopy (Leica SP5, Germany) was used to access images of ECs in different z-stages. Imaris 7.0 software was employed for three-dimensional (3D) reconstruction of confocal images and for analysis of the tubular structure of ECs on micropatterned surfaces.

### Statistical Analysis

Data were represented as mean values ± standard deviation (SD). Statistical analysis was performed by one-way analysis of variance (ANOVA) (OriginPro 8, OriginLab Corporation, USA), followed by LSD or Dunnett post-hoc test for multiple comparisons, where appropriate. A *p*-value less than 0.05 was considered statistically significant.

## Results

### Peptide Immobilization onto Polymer Surface

**Figure 1:**
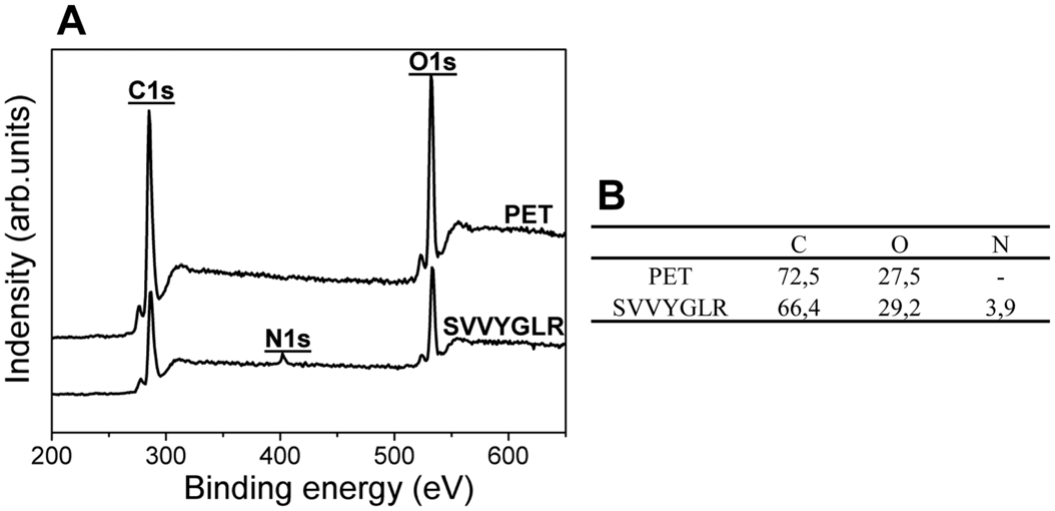
XPS characterization of surfaces. (A) XPS spectrum and (B) experimental atomic composition (%) of PET and PET surfaces grafted with SVVYGLR peptides.

XPS was employed to determine the surface chemical compositions during PET surface modification. The pristine PET surfaces exhibit only C and O elements as expected (Fig. 1). As compared with PET surfaces, PET surfaces grafted with SVVYGLR peptides showed new N1s peaks which appeared at about 399.85 eV (3.9%), corresponding to the successful grafting of SVVYGLR onto the polymer surfaces. Details concerning XPS de-convolution during different steps of surface modification are referred to [32].

### Peptide Micropatterning onto Polymer Surfaces

**Figure 2:**
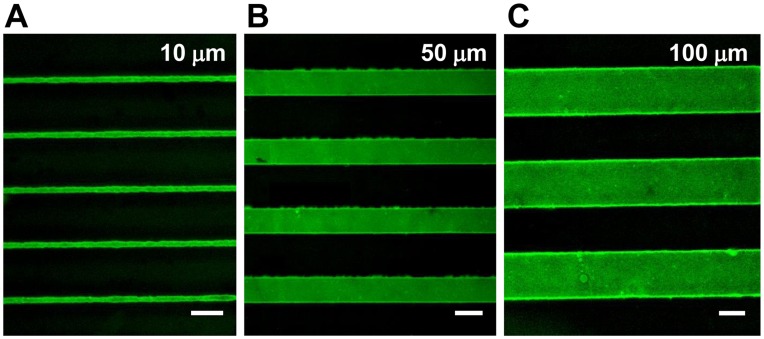
Fluorescent microscopy observation of polymer surfaces micropatterned with fluorescent peptides. The green lines correspond to (A) 10 µm, (B) 50 µm and (C) 100 µm stripes of GDSVVYGLRK-FITC peptides. Scale bars correspond to 50 µm.

Photolithography was used for creating peptide micropatterns onto material surfaces [33]. In this present work, SVVYGLR peptide stripes of three different widths (10, 50 and 100 µm) with the same interspace of 100 µm between the stripes were micropatterned onto PET surfaces. To validate this process, fluorescent peptides (GDSVVYGLRK-FITC) were immobilized onto polymer surfaces for visualization. The polymer surfaces micropatterned with fluorescent peptides are represented in Fig. 2, which confirmed the successful micropatterning of SVVYGLR peptides onto polymer surfaces.

### SVVYGLR Peptides Induced Cell Responses on Homogenous Polymer Surfaces

**Figure 3:**
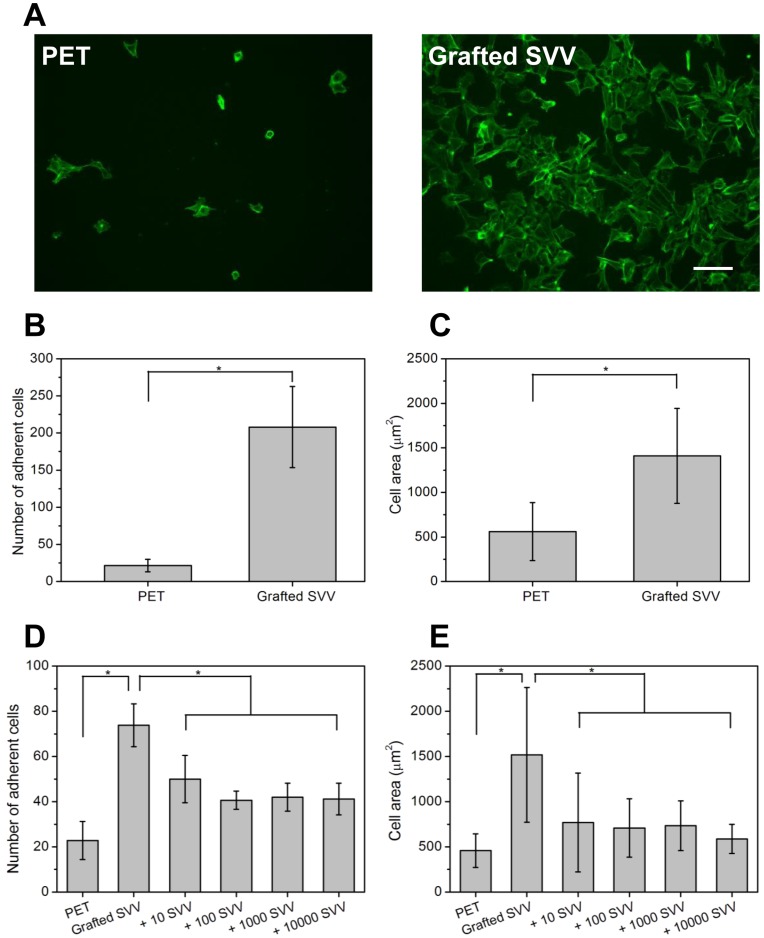
Cell responses induced by grafted and soluble SVVYGLR peptides. (A) EC actin skeleton on PET and PET surfaces grafted with SVVYGLR peptides after 24 h in culture, scale bars are 100 µm. (B) Number of adherent cells and (C) mean cell areas on different surfaces in (A). In competitive experiments, ECs were incubated on SVVYGLR peptides grafted surfaces with presentation of soluble SVVYGLR peptides at different concentrations (10, 100, 1000 and 10000 ng/mL) for 4 h incubation, (D) cell adhesion and (E) cell surface areas on different surfaces. (* *p*<0.01).

At the first step of biological evaluation, SVVYGLR peptides were homogenously grafted onto PET surfaces to study their effect on EC behaviors. The results of cell culture showed that the immobilization of SVVYGLR peptides onto PET induced significant EC adhesion and spreading as compared with pristine PET surfaces (Fig. 3A-C).

To ensure that improved cell adhesion and spreading were mediated by SVVYGLR specific cell receptors, competitive experiments of EC adhesion on SVVYGLR grafted surfaces were evaluated using soluble SVVYGLR peptides in culture medium for 4 h. EC adhesion on SVVYGLR peptide grafted surfaces was reduced in the presence of soluble SVVYGLR peptides over the entire ranges of the soluble peptide concentrations (10, 100, 1000 and 10000 ng/mL), as shown in Fig. 3D. Similar results were also observed in cell spreading levels (Fig. 3E). Similar to pristine PET surfaces, few cells adhered, and most cells were round in shape over the entire range of soluble peptide concentrations. These results suggest that the adhesion and spreading of ECs on the SVVYGLR immobilized surfaces are predominantly mediated by specific cell receptor-SVVYGLR peptide interactions.

The immobilization of SVVYGLR peptides onto PET surfaces induced significant EC adhesion and spreading. ECs on SVVYGLR grafted surfaces formed a confluent monolayer which appeared with typical cobble stone-like morphology after 3 days in culture (Fig. S2). However, neither EC tube formation nor angiogenesis was observed by this approach. Subsequently, the effects of SVVYGLR peptide micropatterning (10 to 100 µm) on EC behaviors as well as angiogenesis (central lumen formation and sprouting) were addressed.

### Micropatterning of SVVYGLR Peptides onto ECs Responses

**Figure 4:**
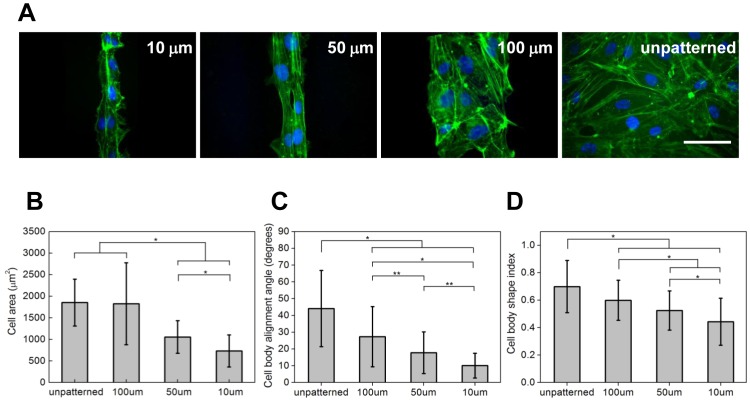
Cell responses induced by the micropatterning of SVVYGLR peptides. (A) ECs on 10 µm, 50 µm and 100 µm SVVYGLR peptide stripes and on unpatterned surfaces. Cells were labeled with phalloidin and nuclei which are represented in green and blue, respectively. Scale bars correspond to 100 µm. Quantification of (B) mean cell areas, (C) cell body alignment angles and (D) cell shape index of ECs on unpatterned surfaces, on 100 µm, 50 µm and 10 µm SVVYGLR peptide stripes (* *p*<0.01; ** 0.01<*p*<0.05).

After 4 h in culture, ECs began to align onto the SVVYGLR peptide patterns regardless of the size of patterns. Fig. 4A illustrated the EC alignment on micropatterned surfaces after 26 h in culture. However, the EC behaviors (cell spreading, alignment, elongation and cell migration) were significantly more regulated on smaller patterns (10 and 50 µm). The quantitative results of cell areas, cell orientation and cell migration on micropatterned and unpatterned surfaces were analyzed.

### EC Spreading, Alignment and Elongation on SVVYGLR Micropatterned Surfaces

Cell areas on unpatterned surfaces and SVVYGLR micropatterns were represented in Fig. 4B. ECs were found to spread most on unpatterned surfaces (mean projected cell areas: 1854±544 µm^2^). Cells seeded on 100 µm SVVYGLR peptide stripes had a similar spreading level as compared with unpatterned surfaces (mean cell areas: 1825±950 µm^2^), while cell spreading was significantly reduced on narrower stripes, with mean projected cell areas of 1056±377 µm^2^ and 732±370 µm^2^ for ECs seeded on 50 µm and 10 µm SVVYGLR peptide stripes, respectively.

Quantitative analyses of cell alignment angles according to the direction of SVVYGLR micropatterns were represented in Fig. 4C. Cells on unpatterned surfaces displayed random orientation, the alignment angles of cell bodies were uniformly distributed, with a mean value of 44.06±22.74°. Cells on patterned SVVYGLR stripes exhibited a strong alignment to the direction of micropatterns (*p*<0.01). For ECs seeded on 100 µm SVVYGLR stripes, mean cell alignment angles decreased to 27.34±17.93° as compared with unpatterned surfaces. The more the pattern width decreased, the more the mean alignment angles decreased: 17.75±12.44° and 10.07±7.36° for ECs on 50 µm and 10 µm SVVYGLR stripes, respectively. Statistical analysis confirmed that there was a main effect of peptide micropattern sizes in driving cell body alignment, and the alignment of cell body became more significant as the pattern size became smaller.

Quantitative analysis of cell body elongation on different surfaces was represented in Fig. 4D. ECs on the unpatterned surface presented a mean cell body shape index of 0.70±0.19. Cells on patterned surfaces showed decreased shape index as compared to the unpatterned surfaces, with mean cell body shape index of 0.59±0.15, 0.52±0.14 and 0.44±0.17 for ECs seeded on 100 µm, 50 µm and 10 µm stripes of SVVYGLR stripes, respectively. The decrease in shape index means that the cell bodies were more elongated on the peptide micropatterns. Statistical analysis showed that the cell elongation on peptide microstripes was more significant as compared with unpatterned surfaces (*p*<0.01), and the elongation on smaller SVVYGLR peptide stripes (50 µm and 10 µm) was more significant (*p*<0.01).

### Geometrical Cues of SVVYGLR Micropatterns Decrease EC Migration

**Figure 5:**
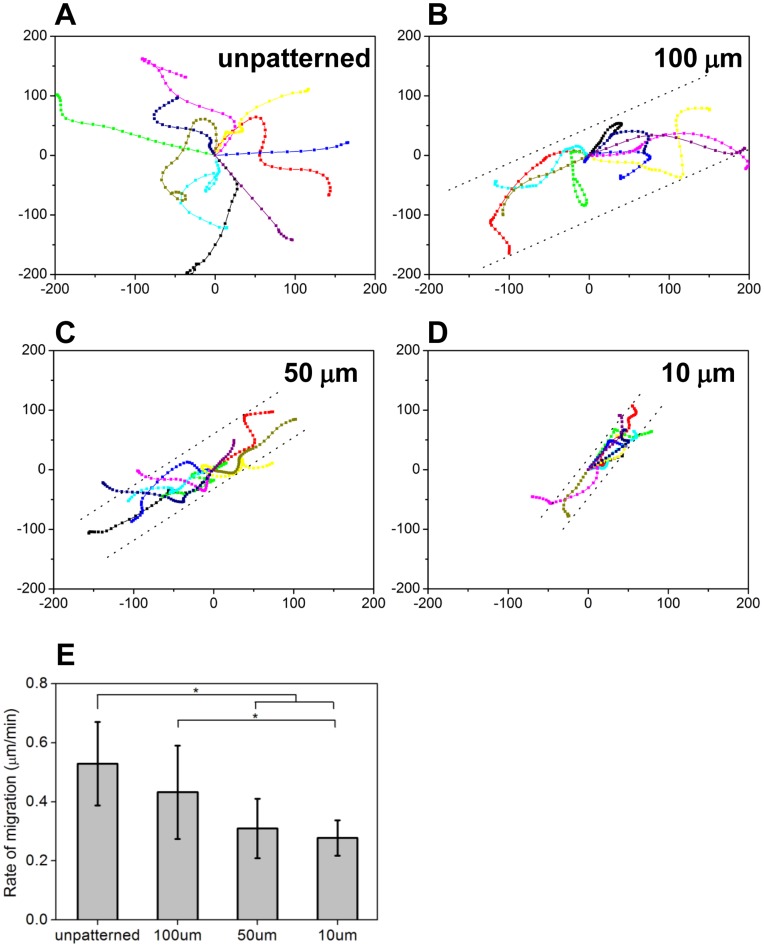
EC migration on micropatterned surfaces. Trajectories of ten ECs on (A) unpatterned surfaces, on (B) 100 µm, (C) 50 µm and (D) 10 µm SVVYGLR peptide stripes. The double dot lines illustrate the peptide micropatterns. (E) Mean rate of EC migration on surfaces of A–D (* *p*<0.01).

EC migration on different surfaces was monitored by time-lapse video microscopy. Fig. 5A–D illustrated the trajectories of cell migration on unpatterned and patterned surfaces during 12 h. ECs on unpatterned surfaces show random directional migration, while the ECs on SVVYGLR patterns exhibit guided migration along the direction of micropatterns. ECs on 10 and 50 µm SVVYGLR stripes have trajectories almost exclusively along the direction of micropatterns.

Total distances traveled by cells were calculated to determine the migration rate of ECs on different surfaces, and the result was summarized in Fig. 5E. There was no significant change in cell migration rate from the unpatterned surfaces to 100 µm SVVYGLR peptide stripes. However, the cell migration rate exhibited a significant decrease on 10 and 50 µm SVVYGLR peptide stripes (*p*<0.01). ECs exhibited a guidance response to micropatterns and restrictedly migrated on the region of micropatterns.

### Geometrical Cues of SVVYGLR Micropatterns Induce Lumen Formation

Furthermore, we investigated the EC morphogenesis on SVVYGLR micropatterned surfaces. Formation of central lumen within these orientated cellular cords was analyzed by confocal microscopy with ECs labeled with a fluorescent cytoplasmic dye (CMFDA) and cell nuclei (DAPI).

**Figure 6:**
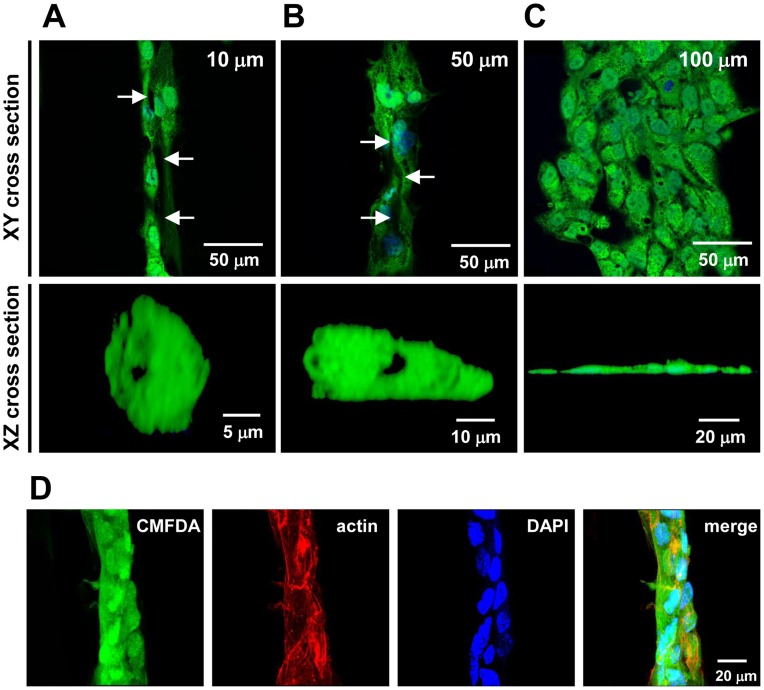
EC tube formation on SVVYGLR micropatterns. Confocal images of ECs seeded on (A) 10 µm and (B) 50 µm SVVYGLR peptide stripes showed a central cavity extending along several cell lengths. The lumen cavity appears as a negatively stained central space when viewed in horizontal (XY) and vertical (XZ) cross section. (C) ECs on 100 µm SVVYGLR stripes remained spread within an adherent monolayer and did not form tubes. Cell staining with Cell Tracker Green (CMFDA) and DAPI were represented in green and blue, respectively. (D) Confocal images of ECs’ cord-like structures on 50 µm SVVYGLR peptide stripes.

The ECs seeded on 10 and 50 µm SVVYGLR peptide stripes underwent morphogenesis and formed capillary tube-like structures (Fig. 6A, B). Confocal images of horizontal and vertical cross sections confirmed the existence of the central lumen, which appeared as a negatively stained central space extending along multiple cell lengths. The ECs also appeared to protrude their cell bodies vertically upwards so that the cytoskeleton system was no longer in a single focal plane characteristic of a spread cell in culture. In contrast, ECs on 100 µm SVVYGLR peptide stripes failed to generate morphogenesis and remained as an adherent, flattened monolayer (Fig. 6C). A vertical cross section of such region showed that ECs remained well spread as a monolayer close to the substrates of patterns. Confocal images in z-stacks also illustrated the cord-like structures of ECs on micropatterns (Fig. 6D for ECs on 50 µm SVVYGLR stripes and Fig. S3 for ECs on 10 µm SVVYGLR stripes).

**Figure 7:**
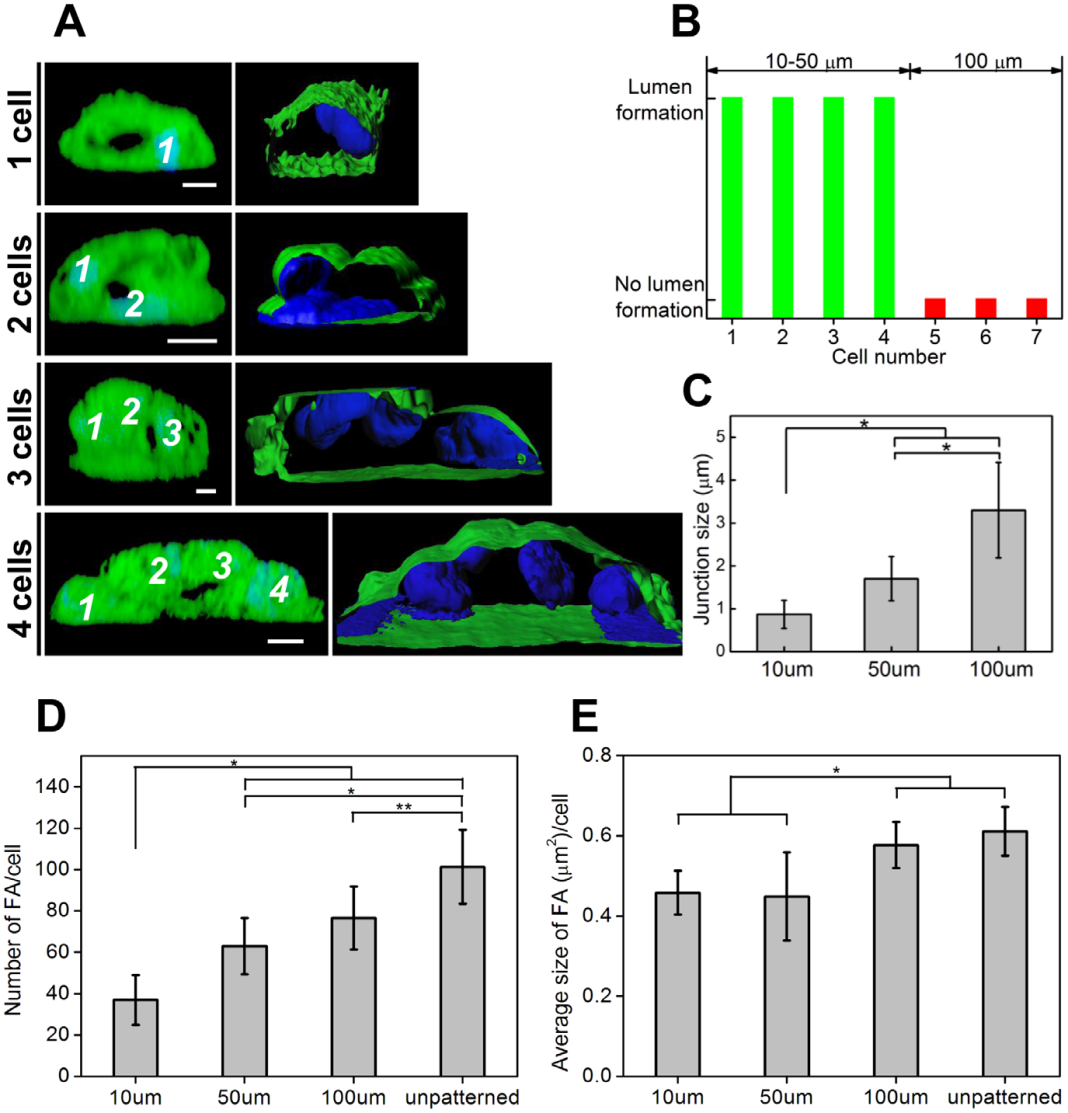
Lumen formation by geometrical cues. (A) Left: vertical confocal image cross sections revealed lumen formation by single-to-four cells, the numbers point out the position of cell nuclei. Right: XZ sections illustrate the lumen surfaces (CMFDA) and the position of cell nuclei (DAPI) corresponding to the images in the left column. Scale bars are 5 µm. (B) Lumen formations can be induced by single-to-four cells on 10 and 50 µm SVVYGLR peptide patterns, but there is no lumen structures formed on 100 µm SVVYGLR peptide patterns. In this last case, the patterns contained more than four cells which cannot support cell-cell reorganization and consequent central lumen formation. (C) Quantification of adherens junctions sizes of cell-cell contacts on SVVYGLR peptide patterns. (D) Number of focal contacts per cell and (E) average size of focal contacts per cell on different surfaces (* *p*<0.01; ** 0.01<*p*<0.05).

Interestingly, vertical cross sections of confocal images showed that the central lumen could be induced by one, two, and three or up to four cells (Fig. 7A). The lumen could be formed by single-to-four cells in the case on 10 or 50 µm micropatterns, but not formed by more than four cells in the case on 100 µm micropatterns (Fig. 7B). Furthermore, we observed that the AJs between ECs cultured on 10 and 50 µm SVVYGLR micropatterns were smaller but more concentrated as compared with those on 100 µm SVVYGLR micropatterns (Fig. S4). Quantitatively, the AJ size was correlated with geometrical constraints applied to ECs by varying the pattern widths (Fig. 7C). Also, we observed a reduced number of focal contacts per cell on patterned surfaces (Fig. 7D). The smaller the micropatterns, the fewer the number of focal contacts per cell on micropatterns. The areas of focal contacts per cell on smaller patterns (10 and 50 µm) were also reduced as comparing with 100 µm micropatterns and unpatterned surfaces (Fig. 7E).

### Tubulogenesis and Formation of Vascular Network

**Figure 8:**
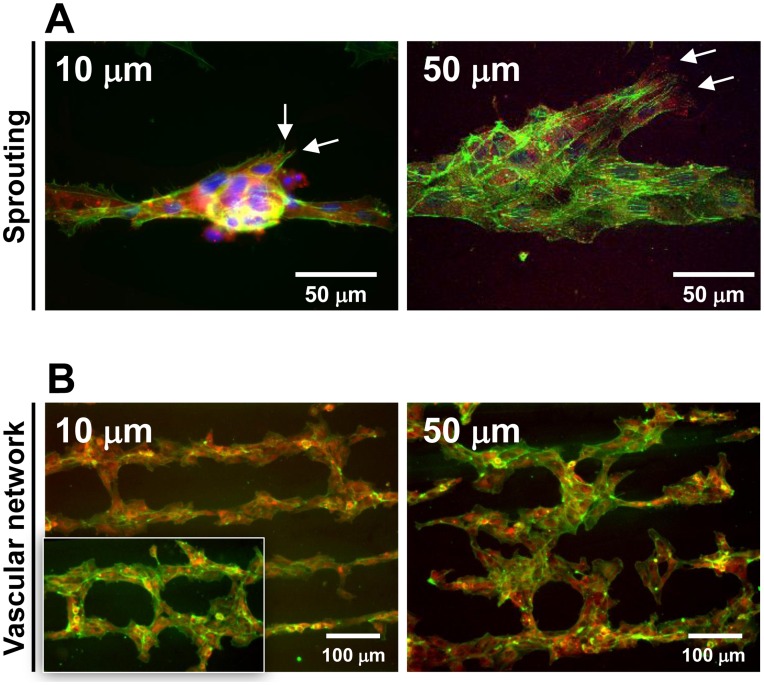
Sprouting and network formation on micropatterned surfaces. (A) Sprouting of ECs and (B) formation of vascular networks on surfaces micropatterned with SVVYGLR peptides. Phalloidin, vinculin and nuclei were stained in green, red and blue, respectively.

Aside from EC tube formation on SVVYGLR peptide micropatterns, immunofluorescent images also revealed ECs sprouting from the pre-formed tubular structure on SVVYGLR micropatterns (Fig. 8A). The sprout cells migrated via filopodial extensions and found receptive ECs from adjacent tubular structures and eventually lead to the formation of vascular networks based on the peptide micropatterned surfaces (Fig. 8B).

## Discussion

Angiogenesis is essential towards the challenge of vascularization in tissue engineering [3]. Although some steps of the angiogenesis process have been identified, the exact mechanism involved in this process is complex and poorly understood. Mimicking angiogenesis will help us both in understanding the process and for its application in tissue engineering.

Many approaches have been developed to modulate angiogenesis with the use of scaffolds made of either natural fibers or polymer fibers [10], [40]. Other assays have described the EC tube formation and promoted angiogenesis based on gel environment [6]. The term lumen is sometimes also used to describe features composed of cells forming circular structures in a nearly two-dimensional (2D) plane [41], [42].

As reported previously, soluble SVVYGLR peptides displayed a similar level of angiogenic activity as VEGF [18], [23]. Here in our work, the mimicking of angiogenesis, *i.e.*, tube formation as well as sprouting of ECs, was induced by micropatterning of SVVYGLR peptides grafted onto 2D polymer surfaces.

Cellular functions on material surfaces are controlled by a complex set of intercellular signaling events, originating from a variety of cell surface receptors. Some of these receptors mediated ECM-cell or cell-cell interactions that are involved in angiogenesis [5], [8]. As reported, the SVVYGLR motif binds the integrin receptors such as α_4_β_1_ [43], α_4_β_7_ [44], α_9_β_1_ [21] and α_v_β_3_ [45], and enhances EC adhesion and migration [18], which are two important processes in angiogenesis.

To ensure that the cell behaviors were mediated by SVVYGLR peptides grafted on PET surfaces, competitive experiments of the effects of soluble SVVYGLR peptides on EC responses were examined. The results showed that grafted SVVYGLR on PET surfaces significantly enhanced EC adhesion and spreading. However, the EC adhesion and spreading on SVVYGLR peptide grafted PET surfaces were reduced in the presence of soluble SVVYGLR peptides over the entire ranges of the soluble peptide density (10, 100, 1000 and 10000 ng/mL) (Fig. 3). These results revealed that the EC adhesion on SVVYGLR peptide immobilized surfaces is predominantly mediated by specific cell receptor-peptide interactions.

When SVVYGLR peptides were micropatterned onto polymer surfaces (Fig. 2), the ECs cultured on the surfaces recognized the micropatterns of SVVYGLR peptides via interaction with integrin receptors and induced EC attachment on the patterns. Subsequently, cell behaviors were guided and regulated by the micropatterned geometrical cues. Microengineered surfaces for cell-based assay were developed to control cell shape and function [25], [26], and some studies reported EC morphogenesis on 2D substrates based on microengineering [19], [27], [28]. In our study, surface micropatterning with angiogenic SVVYGLR peptides was developed to regulate the cellular function and guide EC morphogenesis as well as to induce angiogenesis on polymer material surfaces. The peptide micropatterns described here had identical chemistry, and the ECs were cultured in the same medium. Therefore, the EC behaviors on these micropatterned surfaces differed only in a single parameter: geometrical cues of peptide micropatterns.

The tube-like structures were formed depending on the peptide micropattern sizes. As the results shown in Fig. 4 and Fig. 5, ECs remained in a similar state on larger SVVYGLR peptide stripes (100 µm) as compared with on unpatterned surfaces. However, the cellular functions were significantly more regulated on SVVYGLR micropatterns composed of smaller widths (10 and 50 µm), ECs were restrictedly spread and the cell alignment and elongation were directionally regulated and more significant on narrower patterns (10 and 50 µm) as compared with on larger stripes (100 µm). We also found that ECs preferentially migrated along the direction of micropatterns but the migration rate of ECs was restricted on narrow patterns.

These significant EC responses on narrower micropatterns are important for the remodeling of extracellular matrix, promoting a significant cascade of events resulting in changes in cytoskeletal rearrangement and migration of cells which lead to the assembly of new vessels [8], [19]. These restricted geometries send angiogenic cues to ECs and stimulate reorganization of the EC bodies into tubular structures.

Cell shape changes on the narrow patterns play a critical role in switching between growth and differentiation during angiogenesis [46]. Dike *et al*. showed that the differentiation program, which directs capillary tube formation, can be switched on geometrically [27]. In our study, the small geometry of SVVYGLR patterns (10 and 50 µm) promoted multicellular cell-cell interaction, turning on a tubular differentiation pathway. ECs on smaller patterns underwent vacuole formation by pinocytosis and phagocytosis [47], [48], and these vacuoles coalesce to form lumen in long extensions of capillaries [8]. The vacuoles developed to form continuous tubular structures during several cell lengths along the direction of SVVYGLR peptide patterns (Fig. 6 A, B). In contrast, the ECs on larger patterns remained more spread, migrated and flattened on the patterns and failed to differentiate into tubes (Fig. 6 C).

The results of quantification of ECs’ focal contacts on different surfaces demonstrated that the FA number and size per cell were reduced on smaller patterns (10 and 50 µm) as compared with larger patterns (100 µm) and unpatterned surfaces (Fig. 7D–E). This revealed that the cell-surface adhesions decreased on smaller patterns. These phenomena were reminiscent of cell release from firm contact with the substrates, which is ultimately responsible for promoting tubulogenesis.

Furthermore, we observed that AJs were smaller but more concentrated on smaller peptide micropatterns (Fig. S4 and Fig. 7C). This change in AJ size is probably due to an increase of myosin activity [31]. AJ size and maturation are regulated by geometrical constraints and mechanically influence the ECs cultured on the smaller peptide stripes to form tube-like structure. In this study, we demonstrated that lumen formation is due to micro-geometrical constraints which affect both cell-substrate adhesion and cell-cell adhesion by modulating status of FAs and the correct maturation of AJs, respectively.

Our results suggest that geometrical cues are sufficient to switch ECs into a tubular differentiation program. We demonstrated that the ECs on small patterns self-organized into multi-cellular tubules, and we can adjust tube formation and tube dimensions through the patterns features on the surfaces.

For the first time, we illustrated here in this study the lumen formation by different cell organization on chemical micropatterns: the central lumen of tubular structure can be formed by one, two, and three or up to four cells (Fig. 7). One-cell self-organization into lumen structure was mostly observed on 10 µm SVVYGLR peptide patterns. The lumen formation by two cells is the most common observation during our study. On some SVVYGLR patterns (10 or 50 µm), multiple ECs were stacked on top of each other, two, three or four cells self-organized into the formation of a 3D tubular structure. Lumen structure cannot be observed by more than four cells, such is the case of ECs on larger peptide micropatterns of 100 µm (Fig. 7B).

As reported, the morphogenesis of endothelial cell tube formation can occur via at least two different mechanisms: cell hollowing and cord hollowing [49], [50]. If the cells are arranged in a serial fashion, vacuoles are formed within the cells and coalesce. The fuse of vacuoles gives rise to an intracellular lumen (cell hollowing), and in this process, the lumen is formed by individual cells or chains of cells only one cell thick. We observed this process notably on 10 µm SVVYGLR peptide patterns. Alternatively, if cells are arranged in a paired fashion, they may form a lumen by cord hollowing. In this process, cells assembled into a thin cylindrical cord to create a lumen between cells by the formation and coalescence of vesicles. This tubulogenesis mechanism requires a cell cord two or more cells thick. We also observed this process of cord hollowing and postulate that it is possible to form lumen with 2–4 cells but not more in a 2D culture system. The confocal images presented in our study (Fig. 6–7) suggest that our system of peptide micropatterning supports tubulogenesis through both morphological processes of tube formation.

In our study, angiogenesis activities (tube-like formation and sprouting) were mimicked as the schematic illustrated in Fig. S5. The ECs aligned and restrictedly oriented on the SVVYGLR peptide patterns (Fig. 4, 5), ECs coalesced to form tube-like structures along the length of peptide patterns according to microfeature cues of angiogenic peptides (Fig. 6, 7). EC tube formation on the SVVYGLR peptide patterns played the role of pre-existing vessels, from which EC sprouting can occur and form connections between the adjacent patterns, and the sprouting occurred in parallel to form a vascular network (Fig. 8).

The sprouting angiogenesis requires the tip cells to migrate away from the pre-existing blood vessels [51]–[53]. The adjacent SVVYGLR peptide stripes served as angiogenic cues to each other and trigger the ECs to sprout from pre-formed tubular structures (Fig. 8). The filopodial mode on endothelial tip cells lead cell migration through RhoGTPase, Cdc42 and Rac 1 activation [53], [54]. The stalk cells, comprising the length of the vascular sprout posterior to the tip cells, are highly proliferative and undergo a process of vacuole formation and fusion to form the vessel lumen [51], [53]. The tip cells migrate and eventually find receptive ECs from adjacent vascular structures and eventually lead to the generation of a new vessel (Fig. 8). The angiogenic process occurring in parallel leads to the formation of a vascular network, which should be stabilized by recruitment of mural cells (pericytes and smooth muscle cells) in the future to ensure functional blood vessel network (Fig. S5) [55].

Our work presented here reported the micropattening of SVVYGLR peptides grafted onto polymer surfaces to mimic EC tube formation and vascular network formation, without the surface modification by exogenous growth factors. The EC tube formation can be regulated and guided by micropatterning of SVVYGLR peptides depending on geometrical cues of patterns. EC tubulogenesis and vascular network formation are important towards creating vascularization in tissue engineering and tissue regeneration. Scaffolds sufficiently pre-vascularized *in vitro* could be transplanted *in vivo* and encourage integration with host vasculature. The pre-existing endothelial networks may accelerate the vascularization of large engineered tissues, thus improving the survival of long-term implant of engineered tissues [56]. Modification of polymer surfaces could also be transferred to biodegradable materials for promoting vascularization of scaffold materials. In prospective studies, recruitment of other cell types such as pericytes or smooth muscle cells surrounding the EC tubular structure should be addressed.

### Conclusions

In this study, we reported the EC tubulogeneis and vascular network formation by geometrical cues of angiogenic SVVYGLR peptides on polymer surfaces. It has been shown that EC morphogenesis can be regulated and guided by micropatterning of SVVYGLR peptides depending on the geometrical cues. EC behaviors were significantly more regulated on narrow SVVYGLR micropatterns, ECs were restrictedly spread and the cell orientation and migration were directionally regulated on narrower patterns (10 and 50 µm) as compared with larger stripes (100 µm), resulting in EC morphogenesis into tube formation on the smaller patterns. The central lumen of the tubular structure can be formed by single-to-four cells demonstrating two different processes of tubulogenesis: cell hollowing and cord hollowing. We suggest that EC focal adhesions, AJ size and maturation induced by geometrical constraints participate in the ECs self-assembly into tubular structures. In addition, sprouting of ECs and the formation of vascular network were induced on the surfaces micropatterned with SVVYGLR peptides. The micropatterning of SVVYGLR peptides here provides opportunities for mimicking angiogenesis by avoiding the surface modification by exogenous growth factors. The organization of ECs into tube-like structures and vascular network formation are important toward the fabrication of pre-vascularized tissues, which has great potential applications in tissue engineering and tissue regeneration.

## Supporting Information

Figure S1 **The effects of peptide concentration onto EC alignment.** EC adhesion and alignment on surfaces micropatterned with 50 µm SVVYGLR peptide stripes after 24 h in culture, the concentration of peptide solution was varied from 10^−5 ^M, 10^−4 ^M, 10^−3 ^M to 10^−2 ^M. Scale bar is 100 µm.
(TIF)

Figure S2 **EC adhesion on PET and SVVYGLR grafted surfaces for 3 days.** Cell actin skeletons were represented in green. Scale bar corresponds to 100 µm.
(TIF)

Figure S3 **Confocal images of EC cord-like structure on 10 µm SVVYGLR peptide stripes.**
(TIF)

Figure S4 **EC adherens junctions (AJs) on SVVYGLR peptide micropatterns.** (A) AJs of ECs were obtained from fluorescence staining with antibody against CD31. Scale bars are 20 µm. (B) The AJ size and density on each surface was analyzed by “plot profile” tool in ImageJ: the horizontal axis representing the AJ size and the vertical axis representing the AJ density, respectively.
(TIF)

Figure S5 **Schematic of EC tube formation, sprouting, network formation on micropatterned surfaces and prospective work.**
(TIF)
