# Supplementary figures and images for: Geometrical Microfeature Cues for Directing Tubulogenesis of Endothelial Cells
Source: PLoS One. 2012 Jul 19;7(7):e41163. doi: 10.1371/journal.pone.0041163 (PMC3400641; doi:10.1371/journal.pone.0041163)

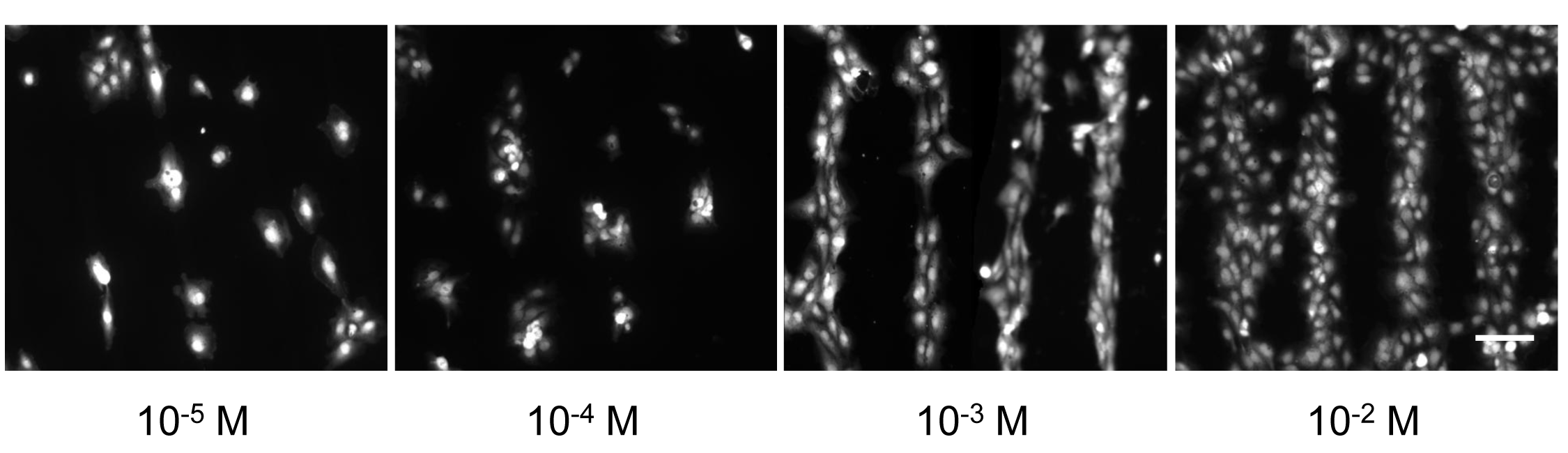

Supplement: Figure S1 — The effects of peptide concentration onto EC alignment. EC adhesion and alignment on surfaces micropatterned with 50 µm SVVYGLR peptide stripes after 24 h in culture, the concentration of peptide solution was varied from 10−5 M, 10−4 M, 10−3 M to 10−2 M. Scale bar is 100 µm. (TIF) [file pone.0041163.s001.tif]

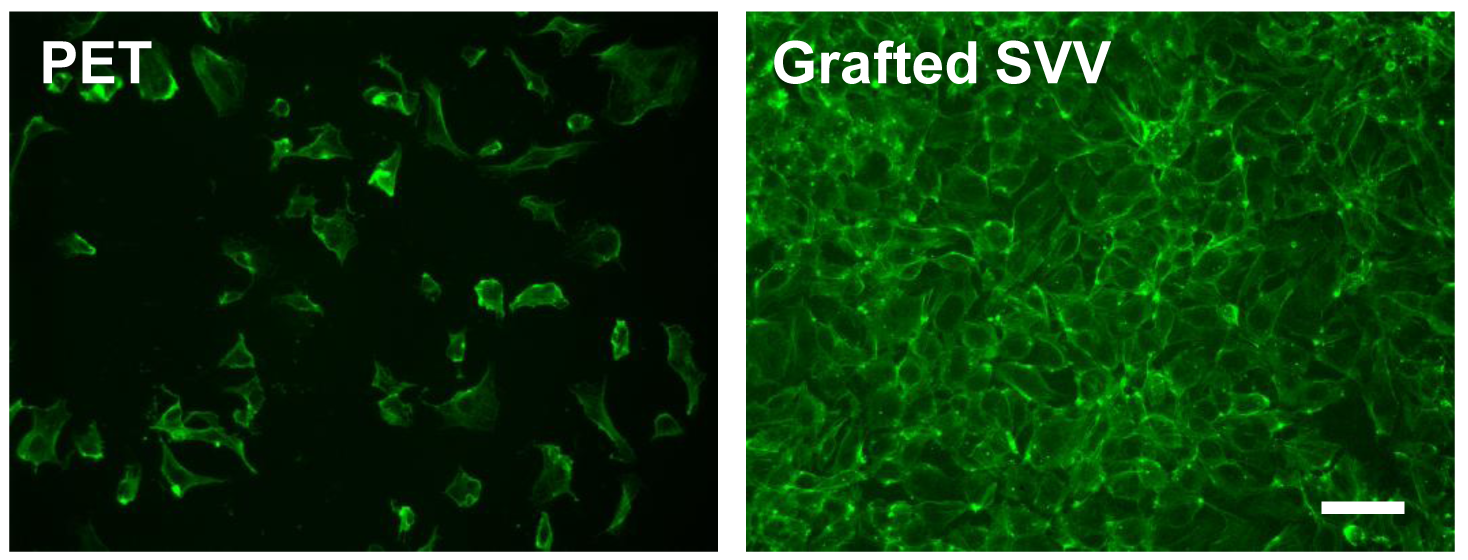

Supplement: Figure S2 — EC adhesion on PET and SVVYGLR grafted surfaces for 3 days. Cell actin skeletons were represented in green. Scale bar corresponds to 100 µm. (TIF) [file pone.0041163.s002.tif]

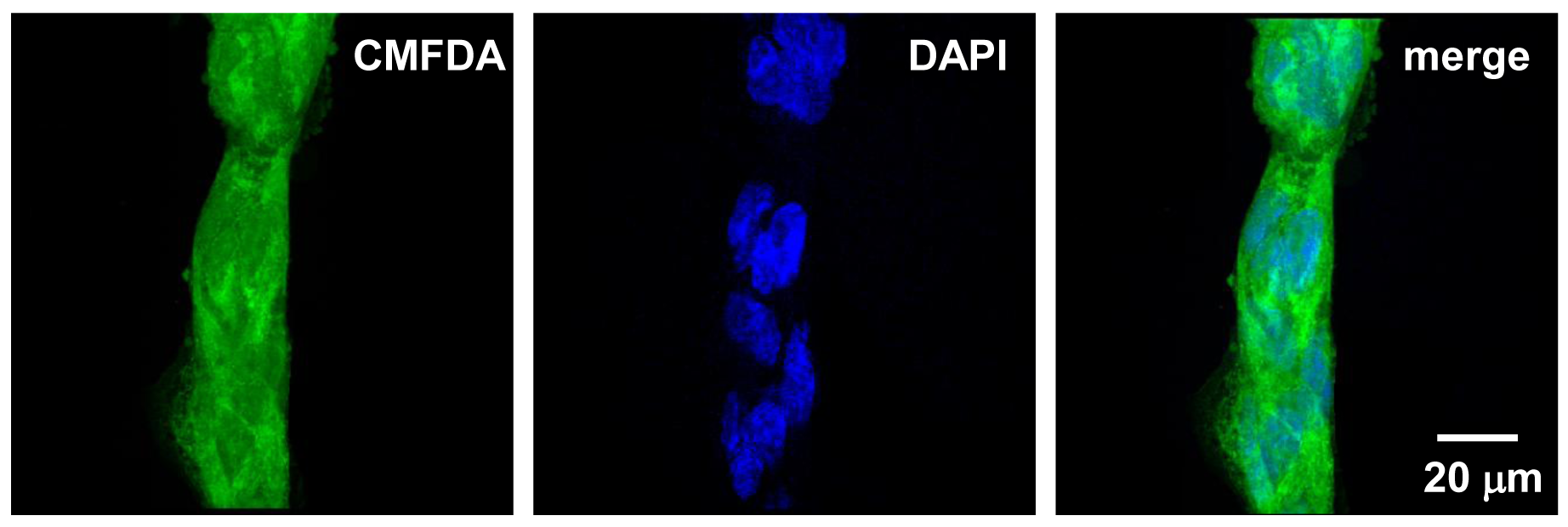

Supplement: Figure S3 — Confocal images of EC cord-like structure on 10 µm SVVYGLR peptide stripes. (TIF) [file pone.0041163.s003.tif]

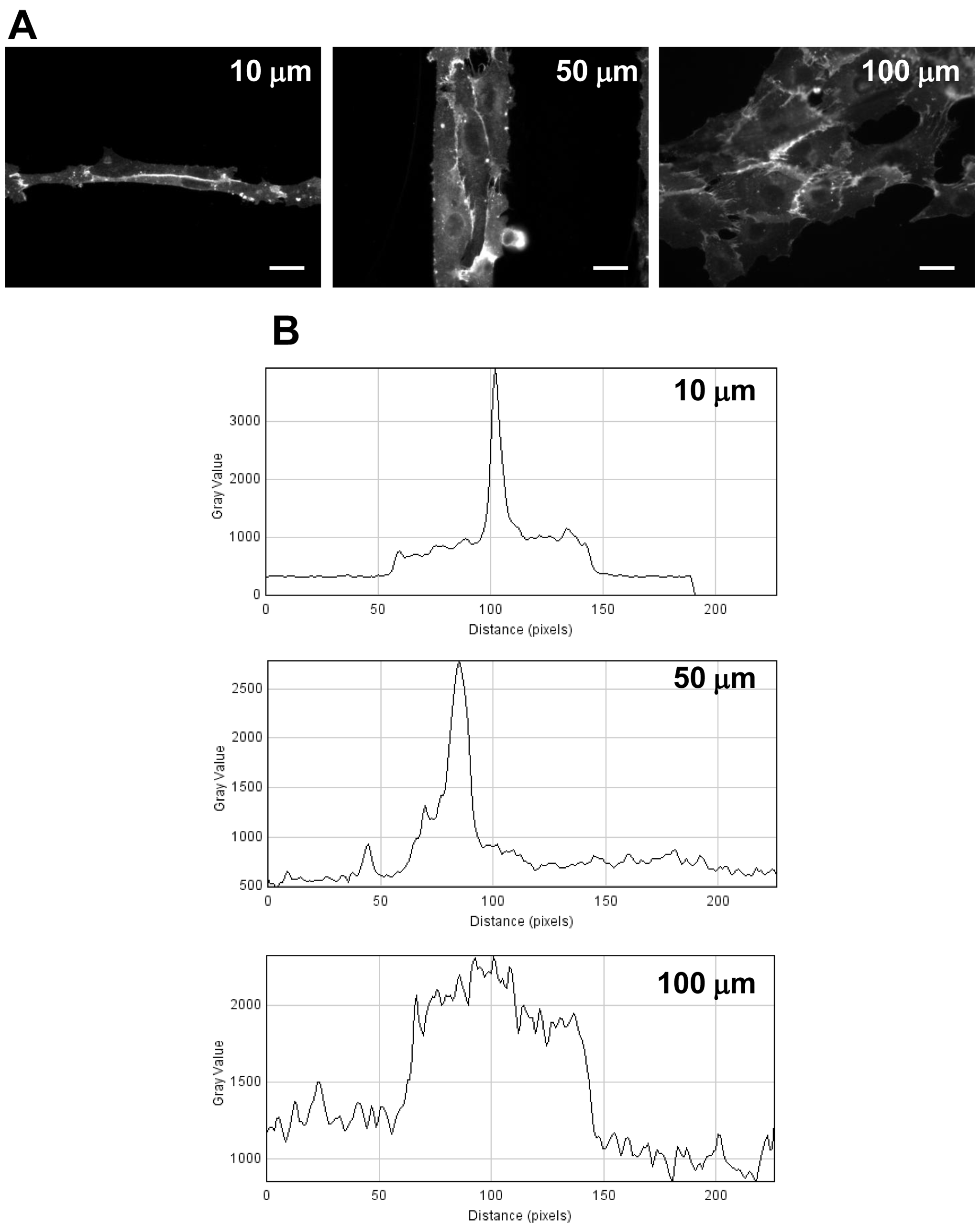

Supplement: Figure S4 — EC adherens junctions (AJs) on SVVYGLR peptide micropatterns. (A) AJs of ECs were obtained from fluorescence staining with antibody against CD31. Scale bars are 20 µm. (B) The AJ size and density on each surface was analyzed by “plot profile” tool in ImageJ: the horizontal axis representing the AJ size and the vertical axis representing the AJ density, respectively. (TIF) [file pone.0041163.s004.tif]

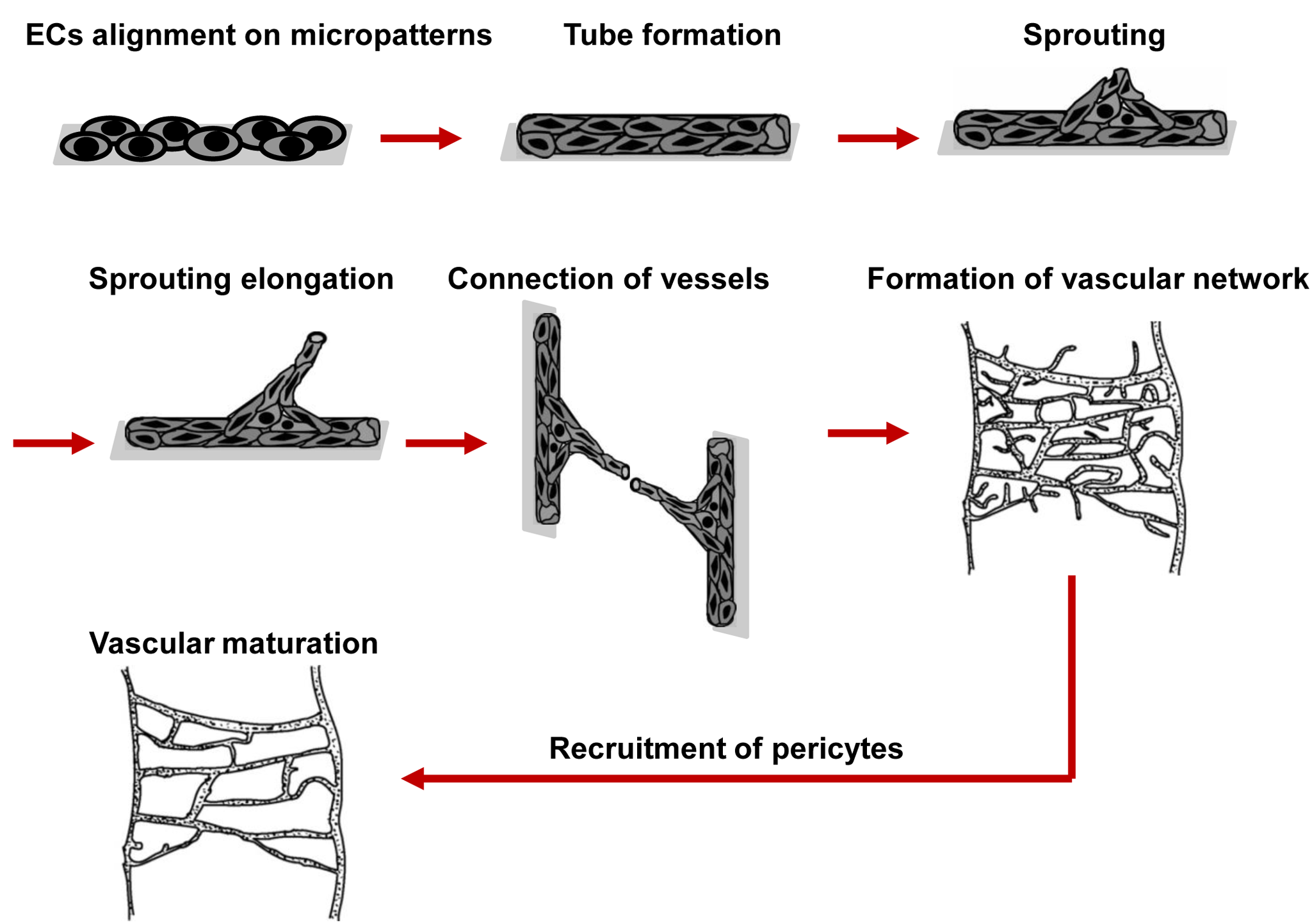

Supplement: Figure S5 — Schematic of EC tube formation, sprouting, network formation on micropatterned surfaces and prospective work. (TIF) [file pone.0041163.s005.tif]
